# Perinatal mortality and other severe adverse pregnancy outcomes associated with treatment of cervical intraepithelial neoplasia: meta-analysis

**DOI:** 10.1136/bmj.a1284

**Published:** 2008-09-18

**Authors:** M Arbyn, M Kyrgiou, C Simoens, A O Raifu, G Koliopoulos, P Martin-Hirsch, W Prendiville, E Paraskevaidis

**Affiliations:** 1 Scientific Institute of Public Health, Brussels, Belgium; 2 European Cooperation on Development and Implementation of Cancer Screening and Prevention Guidelines, IARC, Lyon, France; 3 Department of Obstetrics and Gynaecology, Central Lancashire Teaching Hospitals, Preston, Lancashire; 4 Department of Obstetrics and Gynaecology, University Hospital of Ioannina, Ioannina, Greece; 5 Department of Obstetrics and Gynaecology, Coombe Women’s Hospital, Dublin, Ireland

## Abstract

**Objective** To assess the relative risk of perinatal mortality, severe preterm delivery, and low birth weight associated with previous treatment for precursors of cervical cancer.

**Data sources** Medline and Embase citation tracking from January 1960 to December 2007.

**Selection criteria** Eligible studies had data on severe pregnancy outcomes for women with and without previous treatment for cervical intraepithelial neoplasia. Considered outcomes were perinatal mortality, severe preterm delivery (<32/34 weeks), extreme preterm delivery (<28/30 weeks), and low birth weight (<2000 g, <1500 g, and <1000 g). Excisional and ablative treatment procedures were distinguished.

**Results** One prospective cohort and 19 retrospective studies were retrieved. Cold knife conisation was associated with a significantly increased risk of perinatal mortality (relative risk 2.87, 95% confidence interval 1.42 to 5.81) and a significantly higher risk of severe preterm delivery (2.78, 1.72 to 4.51), extreme preterm delivery (5.33, 1.63 to 17.40), and low birth weight of <2000 g (2.86, 1.37 to 5.97). Laser conisation, described in only one study, was also followed by a significantly increased chance of low birth weight of <2000 g and <1500 g. Large loop excision of the transformation zone and ablative treatment with cryotherapy or laser were not associated with a significantly increased risk of serious adverse pregnancy outcomes. Ablation by radical diathermy was associated with a significantly higher frequency of perinatal mortality, severe and extreme preterm delivery, and low birth weight below 2000 g or 1500 g.

**Conclusions** In the treatment of cervical intraepithelial neoplasia, cold knife conisation and probably both laser conisation and radical diathermy are associated with an increased risk of subsequent perinatal mortality and other serious pregnancy outcomes, unlike laser ablation and cryotherapy. Large loop excision of the transformation zone cannot be considered as completely free of adverse outcomes.

## Introduction

Well organised cervical screening programmes and the appropriate management of screen detected intraepithelial lesions have reduced the incidence of cervical cancer by up to 80%.^1^ Large loop excision of the transformation zone, also known as loop electrosurgical excision, has become the standard treatment for women with cervical precancer in the industrialised world.^2^ ^3^ ^4^ ^5^ Large loop excision has similar failure rates to other treatment methods^6^ but has become the treatment of choice because of other clinical advantages, including the ability to examine the margins of the extirpated transformation zone and thereby assess the completeness of excision, the precise histological diagnosis, and the presence of unexpected glandular or microinvasive disease. The ability to combine diagnosis and treatment in one visit and its low morbidity have also influenced practice.^7^ ^8^ ^9^ Adverse obstetric outcomes after cold knife conisation have been reported.^10^ ^11^^ w1-w3^ Divergent conclusions were drawn regarding the obstetric outcomes for the other excisional treatment procedures,^12^ ^13^^ w4-w7^ whereas ablative methods such as laser ablation or cryotherapy, which destroy cervical tissue, are believed to be free of adverse obstetric risk.^14^^ w8 w9^

As the incidence of cervical intraepithelial neoplasia requiring treatment (that is, grade II or worse) peaks at around the age of 30, any possible effects of such treatment on future childbearing are of particular importance.^15^ In a recent meta-analysis, Kyrgiou et al evaluated a limited number of pregnancy outcomes in women previously treated for cervical intraepithelial neoplasia.^16^ This pooled analysis reported that the risks for preterm delivery among women treated with large loop excision of the transformation zone or cold knife conisation were 1.7 (95% confidence interval 1.2 to 2.4) and 2.6 (1.8 to 3.7) times higher than in untreated women. A significantly increased risk was also noted for low birth weight after both these procedures, for premature rupture of membranes after large loop excision, and for caesarean delivery after cold knife conisation. Preterm delivery, low birth weight, and premature rupture were more common after laser conisation but the differences were insignificant. Laser ablation was not associated with adverse obstetric outcomes. It was concluded that all excisional treatment procedures might be associated with adverse pregnancy outcomes.

The publication of Kyrgiou et al’s meta-analysis has been followed by two small studies^w10 w11^ and four involving large populations.^w12-w15^ This new information, together with data received directly from authors, now allows a new more comprehensive systematic review and meta-analysis with a focus on more serious outcomes like delivery before 32 weeks, birth weight under 2000 g, and perinatal mortality that previous reviews have not been able to analyse.

## Methods

### Studies and interventions, inclusion and exclusion criteria

We included studies with data on severe obstetric or neonatal outcomes in women treated for cervical intraepithelial neoplasia and in a control group of untreated women. Two types of treatment were considered: excisional procedures (cold knife conisation, large loop excision of the transformation zone, and laser conisation) and ablative procedures (laser ablation, cryotherapy, and diathermy).

### Outcome measures

The severe adverse obstetric or neonatal events were perinatal mortality, severe (at less than 32/34 weeks’ gestation) and extreme (<28/30 weeks) preterm delivery, and severe low birth weight (<2000 g, <1500 g, and <1000 g).

### Retrieval of studies and data extraction

Eligible studies published between 1960 and November 2007 were retrieved through a PubMed-Medline and Embase search with the keywords: cervical intraepithelial neoplasia, CIN, cervical cancer, LLETZ, large loop excision of the transformation zone, LEEP, loop electrosurgical excision procedure, cold knife conisation, laser ablation, laser vaporisation, laser conisation, laser excision, pregnancy outcomes, peri-natal mortality, preterm delivery, and low birth weight. We also hand searched references of the retrieved articles and the proceedings of the relevant conferences to identify any articles missed by the initial search and any unpublished data. There was no language restriction. Three authors (MA, MK, and CS) verified inclusion and exclusion criteria independently and reached consensus in case of discordance.

Studies were classified according to type of treatment (excisional or ablative) and by specific treatment. From every included study we extracted or computed the total number of pregnant women treated and not treated for cervical intraepithelial neoplasia and the number of adverse obstetric or neonatal events in both groups. We contacted authors to obtain data on outcomes by particular treatment procedure if they were not provided in the original reports. In addition, we collected data on the study design and matching criteria applied for the selection of a control group of non-treated women.

### Statistical analysis

We calculated the relative risks for each adverse pregnancy outcome in the treated versus untreated women. Studies were separated by type of treatment and further grouped by treatment procedure. We used a random effects model to pool relative risks.^17^ In studies with no events in the treated or control group, we added 0.5 to each cell of the contingency table (continuity correction) to allow calculation of relative risk. We excluded studies with no events in both groups from the meta-analysis. We assessed heterogeneity between studies with Cochrane’s *Q* test and evaluated the percentage of total variation across studies caused by heterogeneity with I^2^.^18^ ^19^ The relative risks for severe adverse pregnancy outcomes were not pooled when there was evidence of significant heterogeneity between studies (P<0.10). In the absence of heterogeneity between groups we computed overall relative risks by weighting the counts of the control group according to the size of the corresponding treated groups for studies that contributed data for multiple procedures.

As severe obstetric outcomes are rare (for instance, the incidence of preterm delivery is less than 1% and several studies have no events in one of the comparison groups), the pooled relative risks can be unstable and influenced by the chosen continuity correction and pooling method.^20^ To test robustness, we applied several alternative methods for pooling (fixed and random effect models, Poisson regression with study population as an offset), weighting of the study estimates (Mantel-Haenszel, reciprocal of the variance), and continuity correction values.^17^ ^21^ ^22^ ^23^

Finally, we pooled the absolute frequency of adverse outcomes after treatment (p_T_) and in the cumulated control populations (p_C_) and derived the number needed to treat to harm (NNTH) as the reciprocal of the risk difference (1/[p_T_−p_C_]). The NNTH reflects the number of women who need to undergo treatment to result in one adverse obstetric event because of the treatment.^24^

We used Stata/SE 9.2 for Windows (StataCorp, College Station, TX) for statistical analysis.

## Results

### Inclusion of studies

We identified seven studies providing information on pregnancy outcomes in women treated for cervical intraepithelial neoplasia that we subsequently excluded as they presented no data on a non-treated control group.^w16-w22^ One further study investigating laser treatment (laser conisation and laser ablation) was excluded as the authors did not provide outcome data for the excisional or the ablative treatment separately.^w23^

We identified 15 studies that fulfilled the eligibility criteria and provided data on perinatal mortality.^w1 w2 w4-w6 w8 w10 w11 w13-w15 w24-w28^ The number of studies that evaluated the other severe pregnancy outcomes was smaller: 11 studies reported on preterm delivery before 34 weeks of gestation^w13 w4 w7 w11-w15 w25 w29 w30^ and five studies on birth weight <2000 g.^w8 w9 w13 w15 w25^ Two studies involved only women treated for carcinoma in situ,^w25 w30^ while the rest included varying degrees of cervical intraepithelial neoplasia. For two of those reports, the original papers did not provide data on procedure specific outcomes, which were obtained directly from the authors.^w13 w14^ For the study by Jakobsson et al procedure specific outcomes were available only for the period 1997-2004.^w14^ We found eight new studies that were not included in the meta-analysis of Kyrgiou et al^16^: six newer reports^w10-w15^ and two older references identified by more comprehensive literature retrieval.^w25 w28^ Reports were written in English, except one that was in Norwegian.^w25^

### Study characteristics

Table 1 describes the characteristics of included studies ranked by year of publication.[Table tbl1] Women were treated by cold knife conisation in nine studies,^w1-w3 w11-w14 w24 w25^ large loop excision of the transformation zone in eight studies,^w4-w7 w10 w11 w13 w14^ and laser conisation in four studies.^w8 w27-w29^ In three studies, women were treated with excision biopsies without further clarification of the specific treatment.^w14 w15 w30^ Pregnancy outcomes after ablative treatment were less often described: two reports after cryotherapy,^w11 w14^ four after laser ablation,^w8 w9 w13 w14^ and only one after diathermy.^w13^

**Table 1 tbl1:** Characteristics of included studies

	Study design	Procedure	Pregnancy outcome	Study size		Inclusion/exclusion criteria	Control of confounding factors
				Treated	Untreated		
Jones, 1979^w1^ (UK)	Retrospective matched cohort	CKC	PM	66	264	Only singleton pregnancies, gestation >28 weeks	Matching for age, parity, social class, date of delivery
Larsson, 1982^w2^ (Sweden)	Retrospective matched cohort	CKC	PM	197	284	PD and late spontaneous abortions due to known factors (uterus bicornis, placenta previa) excluded	Self matching (comparison before and after conisation)
Ludviksson, 1982^w3^ (Sweden)	Retrospective matched cohort	CKC	PD <34 weeks, <30 weeks	83	79	Age <35 years	Matching for age, parity, date of delivery
Kuoppala, 1986^w24^ (Finland)	Retrospective matched cohort	CKC	PM	62	62	Age <40 years	Matching for age, parity, date of delivery
Lund, 1986^w25^ (Norway)	Retrospective matched registry based cohort	CKC	PM, PD ≤28 weeks, LBW <1000 g	251	285	Women born in 1950-4 and pregnancy outcomes 1967-81	Matching for age, period of delivery, and self matching (comparison before and after conisation)
Blomfield, 1993^w5^ (UK)	Retrospective matched cohort	LLETZ	PM	40	80	Only singleton pregnancies	Matching for age, parity, ethnic group, date of delivery
Braet, 1994^w6^ (UK)	Retrospective matched cohort	LLETZ	PM	78	78	Only first singleton viable pregnancies	Matching for age, parity, smoking, date of delivery
Cruickshank, 1995^w7^ (UK)	Retrospective matched cohort	LLETZ	PD <28 weeks	149	298	Only first singleton pregnancies. Gestation ≥20 weeks	Matching for age, parity, smoking, height, social class
Sagot, 1995^w29^ (France)	Retrospective matched cohort	LC	PD <32 weeks	53	59	Non-spontaneous PD excluded	Self matching (comparison before and after conisation)
Bekassy, 1996^w27^ (Sweden)	Retrospective matched cohort	LC (mini-conisation)	PM	250	250	Gestation ≥28 weeks	Matching for age, parity, date of delivery
Forsmo, 1996^w8^ (Norway)	Retrospective matched cohort	LC, LA	PM, LBW <2000 g, <1500 g	LC 65, LA 22	LC 130, LA 44	Only singleton pregnancies	Matching for age, parity, place of delivery
Andersen, 1999^w28^ (Denmark)	Retrospective matched cohort	LC	PM	75	150	Gestation >27 weeks	Matching for age, parity, date, and place of delivery. Other factors (social class, smoking) controlled by logistic regression
El-Bastawissi, 1999^w30^ (US)	Retrospective population based cohort	CKC/LLETZ	PD <34 weeks, <28 weeks	974	7975	Only singleton pregnancies	Frequency matching for age, country of birth. Adjusted for smoking, race, parity, marital status, history of pregnancy termination, by logistic regression
Van Rooijen, 1999^w9^ (Sweden)	Retrospective matched cohort	LA	LBW <2000 g	236	472	Age <35 years	Matching for age, parity, date of delivery
Acharya, 2005^w10^ (Norway)	Retrospective matched cohort	LLETZ	PM	79	158	Age <45 years, gestation >20 weeks, only first pregnancies. Ectopic pregnancies excluded	Matching for age, parity, smoking, date of delivery, previous obstetric history
Samson, 2005^w4^ (Canada)	Retrospective matched cohort	LLETZ	PM, PD <34 weeks	571	571	Age <45 years, gestation >20 weeks, only first pregnancies	Matching for age, parity, smoking, and date and place of delivery
Crane, 2006^w11^ (Canada)	Prospective cohort	CKC, LLETZ, CT	PM, PD <34 weeks	CKC 21, LLETZ 75, CT 36	81	Only singleton pregnancies. Women with known risk factors for PD (previous PD, PD for maternal or fetal reasons) excluded	Adjustment for age, parity, smoking, third trimester bleeding by logistic regression
Klaritsch, 2006^w12^ (Austria)	Retrospective service registry based cohort	CKC	PD <34 weeks	76	29 686	Only singleton deliveries. Women with repeated CIN treatments excluded	None
Bruinsma, 2007^w13^ (Australia)	Retrospective population based cohort	CKC, LLETZ, DT, LA	PM, PD <32 weeks, <28 weeks, LBW <1500 g, <1000 g	CKC 73, LLETZ 69, DT 773, LA 1016	2294	Gestation ≥20 weeks or >400 g	Obstetric antecedents, illicit drug use during pregnancy, major maternal medical condition, birth at study centre, being single, age, referral cytology were significant covariates in logistic regression model for PD. Parity and country of birth were insignificant
Jakobsson, 2007^w14^ (Finland)*	Retrospective population based cohort	CKC, LLETZ, excision, CT, LA	PM, PD <32 weeks, <28 weeks	CKC 92, LLETZ 2690, excision 2064, CT 644, LA 1349	1997-2004: 439 116; 1984-96: 117 429	Age 15-49 years. Only singleton pregnancies	Age, parity, smoking were adjusted for by logistic regression
Sjoborg, 2007^w15^ (Norway)	Retrospective matched cohort	LC/LLETZ	PM, PD <32 weeks, <28 weeks, LBW <1500 g, <1000 g	742	742	Age ≤40 years, gestation ≥16 weeks	Matched for age, parity, plurality. Adjustment for smoking, SE status by logistic regression

CKC=cold knife conisation; LC=laser conisation; LLETZ=large loop excision of transformation zone; excision (NOS)=excision (not otherwise specified); CT=cryotherapy; DT=diathermy; LA=laser ablation; PM=perinatal mortality; LBW=low birth weight; PD=preterm delivery; SE=socioeconomic; CIN=cervical intraepithelial neoplasia.

*For period 1997-2004 data stratified by treatment procedure were obtained from authors; for period 1984-96 only distinction ablative or excisional treatment was available.

Most included studies concerned retrospective cohorts; only one included a prospective cohort.^w11 w25^ In five studies, the control group comprised all women without a history of treatment included in national, regional, or service based birth registries.^w12-w14 w30^ Three studies compared pregnancy outcomes in the same group of women before and after treatment.^w2 w25 w29^ The other studies selected a control population after matching each treated woman with one to four untreated ones for several potential confounding factors such as age, parity, period of birth, smoking status, socioeconomic status, and obstetric antecedents (table 1).[Table tbl1]

### Perinatal mortality

Figure 1 shows the variation in relative risk for perinatal mortality associated with excision of cervical intraepithelial neoplasia.[Fig fig1] This forest plot contains subgroup meta-analyses by treatment procedure. Because of significant heterogeneity between procedures (P=0.031), we have not shown an overall pooled relative risk.

**Figure fig1:**
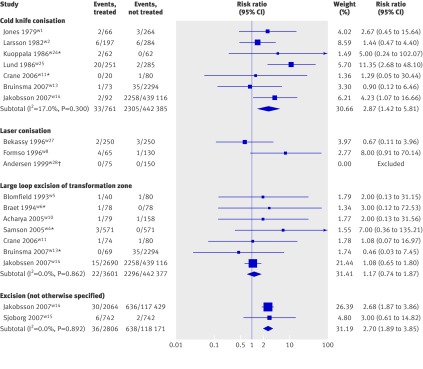
**Fig 1** Meta-analysis of relative risk of perinatal mortality associated with excisional treatment for cervical intraepithelial neoplasia. *0.5 added to each cell of 2×2 contingency table because no cases were found in one of comparison groups. †Excluded because no events in both groups. In subtotals relative risks are pooled by treatment procedure (only computed in absence of significant heterogeneity between studies)

The risk of perinatal mortality was significantly increased in women treated with cold knife conisation (relative risk 2.87, 95% confidence interval 1.42 to 5.81). The Norwegian study showed an outlying high relative risk of 11.35 (2.68 to 48.10).^w25^ Omission of this study still yielded a pooled relative risk that was significantly different from unity (2.08, 1.04 to 4.13).

The risk associated with laser conisation was heterogeneous (I^2^=67%, P=0.082) and therefore not pooled. One study in which mini-conisation was used showed no increase (0.67, 0.11 to 3.96)^w27^ and another showed a substantial increase but did not reach significance (8.00, 0.91 to 70.14).^w8^

Four of seven studies showed a non-significantly increased risk of perinatal mortality in women treated with large loop excision of the transformation zone,^w4-w6 w10^ whereas in three the relative risk was near to or not significantly lower than unity.^w11 w13 w14^ This yielded a pooled relative risk of 1.17 (0.74 to 1.87).

Women whose cervical intraepithelial neoplasia was treated by excision without specification of the procedure showed a significantly increased risk of perinatal mortality.

Although the risk associated with ablative treatment was not increased (fig 2), there was a tendency for increased perinatal mortality in women treated with diathermy (relative risk 1.54, 0.84 to 2.82).

**Figure fig2:**
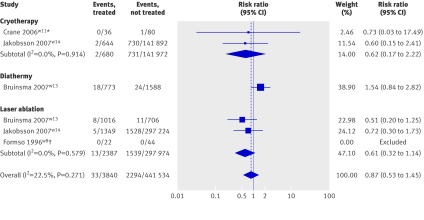
**Fig 2** Meta-analysis of relative risk of perinatal mortality associated with ablative treatment for cervical intraepithelial neoplasia. *0.5 added to each cell of 2×2 contingency table because no cases found in one comparison group, allowing computation of relative risk. †Excluded because no events in both groups. To compute overall relative risk, counts of control groups in reports by Bruinsma^w13^ and Jakobsson^w14^ were weighted proportionally to size of corresponding treated groups to avoid double counting

### Severe and extreme preterm delivery

Severe preterm delivery (gestation <32/34 weeks) was significantly more common after cold knife conisation (pooled relative risk 2.78, 1.72 to 4.51) (table 2).

**Table 2 tbl2:** Meta-analysis of studies comparing outcome of severe preterm delivery (<32/34 weeks) according to treatment for cervical intraepithelial neoplasia

	No (%) of women		Relative risk (95% CI)
	Treated	Not treated	
**Excisional treatment**			
*Cold knife conisation*			
Ludviksson, 1982^w3^* (<34 weeks)	3/83 (3.6)	0/79 (0.0)	6.67 (0.35 to 127.03)
Crane, 2006^w11^* (<34 weeks)	0/21 (0.0)	1/81 (1.2)	1.24 (0.05 to 29.46)
Klaritsch, 2006^w12^ (<34 weeks)	7/76 (9.2)	871/29 686 (2.9)	3.14 (1.55 to 6.38)
Bruinsma, 2007^w13^ (<32 weeks)	4/71 (5.6)	43/2181 (2.0)	2.86 (1.05 to 7.74)
Jakobsson, 2007^w14^ (<32 weeks)	4/92 (4.3)	9542/469 713 (2.0)	2.14 (0.82 to 5.58)
Pooled	18/343 (4.6)	10 457/501 740 (1.6)	2.78 (1.72 to 4.51), P=0.911 (I^2^=0.0%)
*Laser conisation*			
Sagot, 1995^w29^* (<32 weeks)	1/53 (1.9)	0/59 (0.0)	3.33 (0.73 to 16.77)
*Large loop excision of transformation zone*			
Samson, 2005^w4^ (<34 weeks)	7/558 (1.3)	2/558 (0.4)	3.50 (0.73 to 16.77)
Crane, 2006^w11^ (<34 weeks)	3/75 (4.0)	1/81 (1.2)	3.24 (0.34 to 30.47)
Bruinsma, 2007^w13^ (<32 weeks)	1/69 (1.4)	43/2181 (2.0)	0.74 (0.10 to 5.26)
Jakobsson, 2007^w14^ (<32 weeks)	40/2690 (1.5)	9542/469 713 (2.0)	0.73 (0.54 to 1.00)
Pooled	51/3392 (2.0)	9588/472 533 (1.4)	1.20 (0.50 to 2.89), P=0.156 (I^2^=42.7%)
*Excision (not otherwise specified)*			
El-Bastawissi, 1999^w30^ (<34 weeks)	44/974 (4.5)	169/7975 (2.1)	2.13 (1.54 to 2.95)
Sjoborg, 2007^w15^ (<32 weeks)	25/742 (3.4)	6/742 (0.8)	4.17 (1.72 to 10.10)
Pooled	69/1716 (4.0)	175/8717 (1.5)	2.63 (1.41 to 4.89), P=0.154 (I^2^=50.7%)
**Ablative treatment**			
*Cryotherapy*			
Crane, 2006^w11^ (<34 weeks)	1/36 (2.8)	1/81 (1.2)	2.25 (0.14 to 34.98)
Jakobsson, 2007^w14^ (<32 weeks)	11/644 (1.7)	9542/469 713 (2.0)	0.84 (0.47 to 1.51)
Pooled	12/680 (2.2)	9543/469 794 (1.6)	0.88 (0.49 to 1.56), P=0.492 (I^2^=0.0%)
*Diathermy*			
Bruinsma, 2007^w13^ (<32 weeks)	38/760 (5.0)	43/2181 (2.0)	2.54 (1.65 to 3.89)
*Laser ablation*			
Bruinsma, 2007^w13^ (<32 weeks)	23/1005 (2.3)	43/2181 (2.0)	1.16 (0.70 to 1.92)
Jakobsson, 2007^w14^ (<32 weeks)	8/1349 (0.6)	9542/469 713 (2.0)	0.29 (0.15 to 0.58)

*Studies with continuity correction *k*=0.05.

In a small French study, one case of preterm delivery at less then 32 weeks was observed in 53 women who became pregnant after treatment with laser conisation, whereas none was observed in pregnancies before treatment.^w29^

Treatment with large loop excision of the transformation zone was not associated with an increased risk of severe preterm delivery (relative risk 1.20, 0.50 to 2.89) and showed heterogeneous results regarding extreme preterm delivery (table 3).[Table tbl3]

**Table 3 tbl3:** Meta-analysis of studies comparing outcome of extreme preterm delivery (<28/30 weeks) according to treatment for cervical intraepithelial neoplasia

Study*	No (%) of women		Relative risk (95% CI)
	Treated	Not treated	
**Excisional treatment**			
*Cold knife conisation*			
Ludviksson, 1982^w3^†	1/83 (1.2)	0/79 (0.0)	2.86 (0.12 to 69.11)
Lund, 1986^w25^	23/233 (9.8)	1/273 (0.4)	26.95 (3.67 to 198.03)
Bruinsma, 2007^w13^	3/71 (4.2)	20/2,181 (0.9)	4.61 (1.40 to 15.15)
Jakobsson, 2007^w14^	2/92 (2.2)	3938/469 713 (0.8)	2.59 (0.66 to 10.22)
Pooled	29/479 (4.2)	3959/472 246 (0.8)	5.33 (1.63 to 17.40), P=0.130 (I^2^=46.9%)
*Large loop excision of transformation zone*			
Cruickshank, 1995^w7^	4/149 (2.7)	3/298 (1.0)	2.67 (0.60 to 11.76)
Bruinsma, 2007^w13^†	0/69 (0.0)	20/2181 (0.9)	0.76 (0.05 to 12.44)
Jakobsson, 2007^w14^	10/2690 (0.4)	3938/469 713 (0.8)	0.44 (0.24 to 0.82)
*Excision (not otherwise specified)*			
Sjoborg, 2007^w15^	13/742 (1.8)	1/742 (0.1)	13.00 (1.70 to 99.12)
**Ablative treatment**			
*Cryotherapy*			
Jakobsson, 2007^w14^	4/644 (0.6)	3938/469 713 (0.8)	0.74 (0.28 to 1.97)
*Diathermy*			
Bruinsma, 2007^w13^	15/760 (2.0)	20/2181 (0.9)	2.15 (1.11 to 4.18)
*Laser ablation*			
Bruinsma, 2007^w13^	11/1005 (1.1)	20/2181 (0.9)	1.19 (0.57 to 2.48)
Jakobsson, 2007^w14^	3/1349 (0.2)	3938/469 713 (0.8)	0.27 (0.09 to 0.82)

*All <28 weeks except for Ludviksson,^w3^ which was <30 weeks.

†Studies with continuity correction *k*=0.05.

In two studies that used cold knife conisation or another excisional procedure without distinction by procedure, relative risks for severe^w15 w30^ and extreme preterm delivery^w15^ were significantly increased. El-Bastawissi et al used cold knife conisation or large loop excision and observed a relative risk for preterm delivery at <34 weeks of 2.13 (1.54 to 2.95),^w30^ which was intermediate to the pooled relative risks for cold knife conisation (2.78) and large loop excision (1.20). In the other study the relative risks for preterm delivery were 4.17 (1.72 to 10.10) at <32 weeks (table 2) and 13.00 (1.70 to 99.12) <28 weeks (table 3).^w15^[Table tbl3]

Laser ablation or cryotherapy was not associated with higher rates of severe or extreme preterm delivery: relative risks generally were lower but not significantly lower than unity. In one study laser ablation was associated with a significantly lower probability of severe and extreme preterm delivery with relative risks of 0.29 (0.15 to 0.58) and 0.27 (0.09 to 0.82), respectively.^w14^

In one study diathermy resulted in significantly increased rates of both severe (relative risk 2.54, 1.65 to 3.89) and extreme (relative risk 2.15, 1.11 to 4.18) preterm delivery.^w13^

### Severe and extreme low birth weight

Tables 4, 5, and 6 show the effects on birth weight.[Table tbl4] [Table tbl5] [Table tbl6] Three studies that evaluated cold knife conisation, laser conisation, or excision with laser conisation/large loop excision showed a significantly increased risk for birth weights <1500 g^w8 w13 w15^ (table 5). In two Norwegian studies cold knife conisation and excisional treatment (with laser conisation/large loop excision) were associated with extreme low birth weight (<1000 g, table 6).^w15 w25^[Table tbl6]

**Table 4 tbl4:** Meta-analysis of studies comparing outcome of severe low birth weight (<2000 g) according to treatment for cervical intraepithelial neoplasia

Study	No (%) of women		Relative risk (95% CI)
	Treated	Not treated	
**Excisional treatment**			
*Cold knife conisation*			
Bruinsma, 2007^w13^	7/73 (9.6)	77/2293 (3.4)	2.86 (1.37 to 5.97)
*Laser conisation*			
Forsmo, 1996^w8^	7/65 (10.8)	4/130 (3.1)	3.50 (1.06 to 11.53)
*Large loop excision of transformation zone*			
Bruinsma, 2007^w13^	3/69 (4.3)	77/2293 (3.4)	1.29 (0.42 to 4.00)
Pooled excisional	17/207 (8.2)	158/4716 (3.4)	2.47 (1.43 to 4.28), P=0.418 (I^2^=0.0%)
**Ablative treatment**			
*Diathermy*			
Bruinsma, 2007^w13^	53/773 (6.9)	77/2293 (3.4)	2.04 (1.45 to 2.87)
*Laser ablation*			
Forsmo, 1996^w8^	1/22 (4.5)	2/44 (4.5)	1.00 (0.10 to 10.44)
Van Rooijen, 1999^w9^	6/236 (2.5)	13/472 (2.8)	0.92 (0.36 to 2.40)
Bruinsma, 2007^w13^	35/1016 (3.4)	77/2293 (3.4)	1.03 (0.69 to 1.52)
Pooled laser ablation	42/773 (3.4)	92/2809 (3.6)	1.01 (0.71 to 1.45), P=0.980 (I^2^=0.0%)

**Table 5 tbl5:** Meta-analysis of studies comparing outcome of severe low birth weight (<1500 g) according to treatment for cervical intraepithelial neoplasia

Study	No (%) of women		Relative risk (95% CI)
	Treated	Not treated	
**Excisional treatment**			
*Cold knife conisation*			
Bruinsma, 2007^w13^	3/73 (4.1)	41/2293 (1.8)	2.30 (0.73 to 7.25)
*Laser conisation*			
Forsmo, 1996^w8^	5/65 (7.7)	1/130 (0.8)	10.00 (1.19 to 83.84)
*Large loop excision of transformation zone*			
Bruinsma, 2007^w13^	1/69 (1.4)	41/2293 (1.8)	0.81 (0.11 to 5.81)
*Excision (not otherwise specified)*			
Sjoborg, 2007^w15^	17/742 (2.3)	4/742 (0.5)	4.25 (1.44 to 12.57)
Pooled excisional	26/949 (3.9)	87/5458 (1.2)	3.01 (1.38 to 6.56), P=0.311 (I^2^=16.1%)
**Ablative treatment**			
*Diathermy*			
Bruinsma, 2007^w13^	35/773 (4.5)	41/2293 (1.8)	2.53 (1.62 to 3.95)
*Laser ablation*			
Forsmo, 1996^w8^*	0/22 (0.0)	1/44 (2.3)	0.65 (0.03 to 15.39)
Bruinsma, 2007^w13^	20/1016 (2.0)	41/2293 (1.8)	1.10 (0.65 to 1.87)
Pooled laser ablation	20/1038 (1.0)	42/2337 (2.0)	1.09 (0.64 to 1.83), P=0.749 (I^2^=0.0%)

*Study with continuity correction *k*=0.05.

**Table 6 tbl6:** Meta-analysis of studies comparing outcome of severe low birth weight (<1000 g) according to treatment for cervical intraepithelial neoplasia

Study	No (%) of women		Relative risk (95% CI)
	Treated	Not treated	
**Excisional treatment**			
*Cold knife conisation*			
Lund, 1986^w25^	17/251 (6.8)	1/285 (0.4)	19.30 (2.59 to 144.01)
Bruinsma, 2007^w13^	2/73 (4.1)	41/2293 (1.8)	1.53 (0.38 to 6.21)
*Large loop excision of transformation zone*			
Bruinsma, 2007^w13^*	0/69 (0.0)	41/2293 (1.8)	0.39 (0.02 to 6.35)
*Excision (not otherwise specified)*			
Sjoborg, 2007^w15^	11/742 (1.5)	1/742 (0.1)	11.00 (1.42 to 84.99)
**Ablative treatment**			
*Diathermy*			
Bruinsma, 2007^w13^	11/773 (1.4)	41/2293 (1.8)	0.80 (0.41 to 1.54)
*Laser ablation*			
Bruinsma, 2007^w13^	10/1016 (1.0)	41/2293 (1.8)	0.55 (0.28 to 1.09)
Pooled ablative	21/1789 (1.2)	82/4586 (1.8)	0.67 (0.41 to 1.07), P=0.448 (I^2^=0.0%)

*Study with continuity correction *k*=0.05.

Laser ablation was not associated with increased risks for very low birth weight (table 6), while a significantly higher rate of birth weights of <2000 g (table 4) and <1500 g (table 5) was observed in women treated with diathermy.^w13^[Table tbl4] [Table tbl5]

### Robustness of pooled relative risks

Treatment of cervical intraepithelial neoplasia with large loop excision resulted in a non-significantly increased risk of perinatal mortality (1.17, 0.74 to 1.87) (fig 1).[Fig fig1] Three of the seven studies, however, showed no counts in one of the comparison groups,^w4 w6 w13^ necessitating the use of a continuity correction (κ=0.5). Table 7 shows the results of alternative models for combining relative risks and different continuity corrections. All models and continuity corrections resulted in similar pooled estimates, underlying the robustness of the meta-analysis. Similar pooled relative risks for perinatal mortality were also obtained for the other excisional methods (data available from authors).

**Table tbl7:** 7 Relative risk (95% confidence interval) for perinatal mortality in women treated with large loop excision of transformation zone for cervical cancer precursors versus women not treated. Results obtained with different models and methods for continuity correction in instances of zero cases of perinatal mortality in one of comparison groups

Model	Continuity correction*			
	1	2	3	4
Mantel-Haenszel:				
Fixed effect	1.21 (0.77 to 1.91)	1.19 (0.74 to 1.89)	1.19 (0.74 to 1.89)	1.20 (0.76 to 1.91)
Random effect	1.18 (0.74 to 1.88)	1.14 (0.70 to 1.84)	1.14 (0.70 to 1.85)	1.20 (0.75 to 1.93)
Inverse variance:				
Fixed effect	1.18 (0.74 to 1.88)	1.14 (0.70 to 1.84)	1.14 (0.70 to 1.85)	1.20 (0.75 to 1.93)
Random effect	1.17 (0.74 to 1.87)	1.14 (0.70 to 1.84)	1.14 (0.70 to 1.85)	1.20 (0.75 to 1.93)
Poisson regression (fixed effect)†	1.18 (0.98 to 1.41)			

*1: adding constant *k* to each cell in 2×2 contingency table (*k*=0.05); 2: same as (1) with *k*=0.01; 3: *k* computed from reciprocal of size of non-treated group; 4: *k* computed empirically from studies without zero cases of perinatal mortality.

†No continuity correction applied.

Too few studies evaluating other outcomes were available to test the robustness of the pooled estimates.

### Obstetric harm after treatment

We pooled the absolute frequency of adverse obstetric outcomes after treatment (p_T_) and in the cumulated control populations (p_C_) and derived the number needed to treat to observe obstetric harm in one treated woman (NNTH) (table 8).[Table tbl8] We excluded the study of Lund et al^w25^ because of outlying relative risks that we considered were not representative for the other studies.

**Table 8 tbl8:** Meta-analysis of adverse obstetric outcomes in treated women (by procedure) and in non-treated control populations, with pooled frequency of obstetric events and number needed to treat to observe harm (NNTH)

Outcome and procedure	No of studies	No of events	No (%, 95% CI)	NNTH
**Perinatal mortality **				
Cold knife conisation	6	13	510 (2.2, 1.5 to 2.9)	71
Laser conisation	3	6	390 (2.3, 0.8 to 3.9)	67
Large loop excision	7	22	3601 (1.0, 1.0 to 1.1)	500
Radical diathermy	1	18	773 (2.3, 2.3 to 2.3)	67
Control	14	6325	1 055 673 (0.8, 0.6 to 1.0)	—
**Preterm delivery <32/34 weeks**				
Cold knife conisation	5	18	343 (4.6, 3.0 to 6.1)	30
Laser conisation	1	1	53 (1.9, 1.8 to 2.0)	167
Large loop excision	4	51	3392 (2.0, 1.8 to 2.2)	143
Radical diathermy	1	38	760 (5.0, 5.0 to 5.0)	27
Control	9	10 634	500 440 (1.3, 0.9 to 1.7)	—
**Preterm delivery <28/30 weeks**				
Cold knife conisation	3	6	246 (2.5, 1.3 to 3.7)	53
Large loop excision	3	14	2908 (1.0, 0.0 to 2.7)	250
Radical diathermy	1	15	760 (2.0, 2.0 to 2.0)	71
Control	5	3962	473 013 (0.6, 0.1 to 1.0)	—
**Low birth weight <2000 g**				
Cold knife conisation	1	7	73 (9.6, 2.8 to 16.3)	16
Laser conisation	1	7	65 (10.8, 3.2 to 18.3)	14
Large loop excision	1	3	69 (4.3, <0.0 to 9.2)	106
Radical diathermy	1	53	773 (6.9, 5.1 to 8.6)	29
Control	4	96	2939 (3.4, 3.0 to 3.8)	—
**Low birth weight <1500 g**				
Cold knife conisation	1	3	73 (4.1, <0.0 to 8.7)	36
Laser conisation	1	5	65 (7.7, 1.2 to 14.2)	16
Large loop excision	1	1	69 (1.4, <0.0 to 4.3)	670
Radical diathermy	1	35	773 (4.5, 3.1 to 6.0)	31
Control	4	47	3209 (1.3, 0.5 to 2.2)	—
**Low birth weight <1000 g**				
Cold knife conisation	1	2	73 (2.7, <0.0 to 6.5)	54
Large loop excision	1	0	69 (0.0, 0.0 to 0.0)	—
Radical diathermy	1	11	773 (1.4, 0.6 to 2.3)	191
Control	2	42	3035 (0.9, <0.0 to 2.6)	—

We estimated that previous treatment with cold knife conisation, laser conisation, or diathermy would result in about one perinatal death in every 70 pregnancies. After large loop excision of the transformation zone, however, we estimate only two perinatal deaths in 1000 pregnancies. Severe and extreme preterm delivery and low birth weight were common (NNTH often <60) after cold knife conisation and diathermy but rare after large loop excision (NNTH (delivery <32-34 weeks, birth weight <2000 g) >100 or NNTH (birth weight <1500 g) >500).

## Discussion

The current meta-analysis shows that, among all the excisional methods used in the treatment of cervical intraepithelial neoplasia, cold knife conisation is consistently associated with serious adverse pregnancy outcomes. Laser conisation increased the risk of perinatal mortality and very low birthweight infants when we excluded from the analysis one study that modified the technique and excised a substantially smaller amount of tissue.^w27^

Several new studies and reviews have recently been published on outcomes of pregnancy after treatment for cervical intraepithelial neoplasia.^w12-w15 ^25 26 27 These new data increased the sample size and statistical power and enabled us, for the first time, to address the rarer and more serious outcomes such as perinatal mortality, severe and extreme preterm delivery (<28 weeks), and very low birth weight (<2000 g). These outcomes have a considerable impact not only on the mothers and babies concerned but also on the health budget for neonatal intensive care. 

The studies included in the earlier meta-analysis of Kyrgiou et al revealed an increased risk for preterm delivery and low birth weight associated with large loop excision,^16^ but in our meta-analysis we found that it did not significantly affect the more serious adverse obstetric events. Both meta-analyses corroborate the conclusion that ablation with laser has no effects on obstetric outcomes. The recent study by Jakobsson et al reported similar findings for cryotherapy.^w14^ Bruinsma et al reported that radical diathermy, an aggressive ablative method that destroys tissue to a depth of about 1 cm, was associated with perinatal mortality, extreme preterm delivery, and severe low birth weight, which was of the same order of magnitude as seen with treatment with cold knife conisation.^w13^ The significantly decreased risk of serious pregnancy outcomes in women treated by laser ablation in the study of Jakobsson et al,^w14^ is probably because of the preferential use of this procedure in Finland for women with small or less severe lesions and at low risk of preterm delivery (P Nieminen, personal communication, 2008).

### Biological mechanisms

Removal or destruction of part of the cervix might compromise its function, leading to lack of mechanical support in a future pregnancy and subsequent premature rupture of membranes and preterm delivery. A reasonable hypothesis would be that the degree of obstetric morbidity noted between therapeutic procedures might be related to the amount of the cervical tissue removed or destroyed, which is less pronounced with ablative techniques such as laser ablation and cryotherapy. Several investigators have described a positive association between depth of excision and risk of adverse obstetric events.^w4 ^28 29 30 The proportion of the total cervical volume or endocervical canal removed might be more important than the actual depth of excision. Inevitably, the knife excises, on average, more tissue than the loop, while loop excisions might vary considerably from superficial and low volume to deep and large volume cones. The retrospective studies included in this meta-analysis presented wide variations in the loop sizes used and consequently the cone volume removed, which probably explains the wide range of relative risks (from 0.46 to 7.00) and the non-significant pooled effect of loop excision on perinatal mortality. On the other hand, the lack of any adverse effects with the use of laser ablation and cryotherapy might be explained by the nature of the destruction of tissue that extends at a rather steady depth, which should be about 5 mm,^6^ ^31^ whereas in loop excision the excision is usually deeper at the centre than at the edges.^32^ Others suggested that pathophysiological mechanisms might also be mediated by the different composition of the quality of collagen in the regenerated cervix^33^ or other immunological factors, such as impairment of the defence mechanisms and alteration of the cervicovaginal flora.^34^

### Alternative explanations

One of the major questions is whether the observed differences in the frequency of adverse pregnancy outcomes can be explained by other factors. As comparison groups (treated for cervical intraepithelial neoplasia with a particular procedure versus non-treated) were non-randomised, effects and effect sizes cannot be attributed with certainty to the treatment alone.^35^ Women with cervical intraepithelial neoplasia are known to have demographic, behavioural, and sexual characteristics that increase their risk of adverse obstetric outcomes. Bacterial vaginosis, for instance, is associated with premature rupture of membranes and is found more often in women with cervical intraepithelial neoplasia than in the general screening population.^36^ ^37^ ^38^ In most studies, the reference group was drawn from the general obstetric population with partial adjustment for confounding factors by matching for age, smoking status, parity, etc. One exception was the study by Bruinsma et al, in which both treated and non-treated women were drawn from women referred for assessment of cervical cytological abnormalities and which showed the lowest relative risk of perinatal mortality associated with cold knife conisation or large loop excision of the transformation zone (see fig 1).^w13 ^29 [Fig fig1]

A study from Norway^w25^ showed that women exposed to risk factors for cervical intraepithelial neoplasia are also at risk of serious pregnancy outcomes. Pregnant women who were subsequently diagnosed with cervical carcinoma in situ showed a risk of perinatal mortality, before cold knife conisation, that was already 21% higher than in women who had never been treated. Pregnancies after cold knife conisation were associated with a relative risk of 11.4. When we accounted for the increased risk before conisation, the adjusted relative risk for perinatal mortality was 9.4.

Potential inflation of the relative risks due to the choice of a reference group, that does not share other risk factors for adverse pregnancy outcomes, was also mentioned by Sadler et al.^29^ In their study in New Zealand, treated women and women in the non-treated comparison group were both recruited from a colposcopy clinic. The resulting relative risk for preterm delivery associated with large loop excision was 1.30 (0.89 to 1.88), which was lower than the pooled relative risk.^16^ These data indicate that factors other than the treatment of cervical intraepithelial neoplasia are contributing to the risk of preterm delivery and studies that select their controls from the general population would therefore be biased in favour of detecting an effect.

Moreover, women who require treatment for cervical intraepithelial neoplasia are selected for one treatment or another on the basis of several important characteristics that are likely to affect the chance of subsequent morphological damage to the cervix. These include size, severity, and site of the lesion, anatomical characteristics of the transformation zone, and suspicion of glandular neoplasia or microinvasive disease.^39^ In general, ablation is used to treat areas that are smaller and less severe, whereas excisional treatments are used for the former indication but also when there is a suspicion of invasion, a larger area, or transformation zones extending deep in the endocervical canal. This means that there is already an inherent bias towards removal of larger areas of the cervix with excisional treatments, which one would expect to be associated with a worse obstetric outcome in the future.

Although all these factors could explain observed effects, the fact that the size and direction of the relative risks were consistent throughout the studies, with adjustment for various factors, supports the conclusion that cold knife conisation, laser conisation, and radical diathermy do increase the risk of serious adverse pregnancy outcomes.

Moreover, study design characteristics and quality parameters (prospective *v* retrospective design, practice *v* population based selection of patients, adequacy of control for confounding factors, procedure for matching treated to non-treated patients) did not explain the heterogeneity between studies (data of meta-regression not shown but available from authors).

### Implications for practice

Whether there is a critical threshold in the amount of tissue excised or destroyed that determines obstetric morbidity and success of treatment in terms of recurrent cervical intraepithelial neoplasia or cancer are key questions that remain to be answered. Having a clear understanding of this relation would be useful in guiding clinical decision making. Three recent studies have shown that treated women are still at higher risk than the general population for developing subsequent invasive cervical cancer, even many years after treatment,^40^ ^41^ ^42^ and some gynaecologists warn that less aggressive treatments might increase this risk.^43^ Evidence indicates that testing for human papillomavirus can help with the follow-up of women after treatment for cervical intraepithelial neoplasia. In particular, because of its high negative predictive value, it can clearly identify those women who are at low risk of residual or recurrent disease,^44^ ^45^ ^46^ and this could be used to alleviate reservations about shifting to less aggressive treatment practices. Moreover, optimal triage and diagnostic procedures should be developed that select only those progressing cases that need aggressive treatment.^45^ ^47^ Introduction of prophylactic vaccines for human papillomavirus will result in a considerable decrease in the incidence of cervical cancer and precursor lesions requiring treatment, which will subsequently reduce the adverse obstetric consequences.

### Conclusions

All excisional procedures used to treat cervical intraepithelial neoplasia seem to be associated with adverse obstetric morbidity, but among these, only cold knife conisation is associated with a significantly increased rate of severe outcomes. The risk of serious obstetric morbidity associated with large loop excision of the transformation zone was not significantly different from unity, though we cannot exclude the possibility of any increased risk. Loop excisions that remove large amounts of cervical tissue probably have the same effect as knife cone biopsies. Most loop excisions in young women with fully visible transformation zones need to be only 1 cm deep, and this should protect against serious obstetric outcomes. Given the design of published observational studies, observed differences in perinatal mortality and severe premature delivery in treated versus non-treated women cannot be ascribed solely to treatment. Moreover, the precise conditions that determine the oncological and reproductive health outcomes are insufficiently known and require further research. Nevertheless, women of reproductive age should be informed about the potential impact on future pregnancies. Gynaecologists should tailor the management of young woman to minimise possible adverse obstetric outcomes at the same time as minimising residual disease rates.

**What is already known on this topic**

Women treated for cervical intraepithelial neoplasia with excisional procedures have an increased risk of preterm delivery and low birth weight in future pregnancies

**What this study adds**

Women who become pregnant after treatment for cervical intraepithelial neoplasia with cold knife conisation and radical diathermy have an increased risk of perinatal mortality, severe preterm delivery, and extreme low birthweight infants
The commonly used loop excision is associated with mild but not with severe obstetric morbidity

## Notes

We thank F Bruinsma (Mother and Child Health Research, La Trobe University, Carlton, Victoria, Australia) and M Jakobsson (department of obstetrics and gynaecology, University Hospital, Helsinki, Finland) for the provision of additional procedure specific data; and Tini Van Dijk (Norwegian Cancer Registry) for translation help.

Ethical approval: Not required.

Provenance and peer review: Not commissioned; externally peer reviewed.

## Notes

**Cite this as: ***BMJ* 2008;337:a1284
